# Factors associated with testing positive for SARS-CoV-2 and evaluation of a recruitment protocol among healthcare personnel in a COVID-19 vaccine effectiveness study

**DOI:** 10.1017/ash.2024.44

**Published:** 2024-04-16

**Authors:** Morgan M. Millar, Jeanmarie Mayer, Jacob Crook, Kristina M. Stratford, Tavis Huber, Matthew H. Samore

**Affiliations:** 1 Division of Epidemiology, Department of Internal Medicine, University of Utah, Salt Lake City, UT, USA; 2 Department of Veterans Affairs, Salt Lake City Healthcare System, Salt Lake City, UT, USA

## Abstract

**Objective:**

The objective of this study was to determine factors associated with testing positive for SARS-CoV-2 among healthcare personnel. Secondary objectives were to assess representativeness of recruited participants and the effectiveness of a multiple-contact protocol for recruiting healthcare personnel in this COVID-19 study.

**Design:**

Survey study, conducted as part of an observational test-negative study of COVID-19 vaccine effectiveness.

**Setting:**

University of Utah Health system, including both inpatient and outpatient facilities.

**Participants:**

Clinical and non-clinical healthcare personnel at University of Utah Health. 1456 were contacted and 503 (34.5%) completed the survey. Cases were all eligible employees testing positive for COVID-19, with 3:1 randomly selected, matched controls (test negative) selected weekly.

**Methods:**

Online survey.

**Results:**

Significant differences in the demographics of participants and the source population were observed; e.g., nursing staff comprised 31.6% of participants but only 23.3% of the source population. The multiple-contact recruitment protocol increased participation by ten percentage points and ensured equal representation of controls. Potential exposure to illness outside of work was strongly predictive of testing positive for SARS-CoV-2 (OR = 3.74; 95% CI: 2.29, 6.11) whereas potential exposure at work was protective against testing positive (OR: 0.51, 95% CI: 0.29, 0.88).

**Conclusions:**

Carefully designed recruitment protocols increase participation and representation of controls, but bias in participant demographics still exists. The negative association between potential workplace exposure and positive test suggests testing bias in the test-negative design. Healthcare personnel’s potential exposures to COVID-19 outside of the workplace are important predictors of SARS-CoV-2 seropositivity.

## Introduction

Early into the COVID-19 pandemic, concerns arose about the risks of exposure in healthcare facilities. Once the United States Food and Drug Administration gave Emergency Use Authorization to the two mRNA vaccines in December 2020,^1^ healthcare personnel were prioritized for vaccination due to their potentially higher risk of exposure and transmission.^2^ Early vaccination of U.S. healthcare personnel provided opportunities for large sample, real-world evaluations of vaccine effectiveness^3^ and investigations of preventive behaviors and risk factors for healthcare-associated SARS-CoV-2 infections. Such information can aid in the prevention of future healthcare-associated infections.

As a participating site in a COVID-19 mRNA vaccine effectiveness study, in 2020–2021 we recruited University of Utah (UU) healthcare personnel to participate in a test-negative case-control study and surveyed participants about potential COVID-19 exposures and protective behaviors at work and in the community. The validity of such observational studies to evaluate COVID-19 vaccine effectiveness depends in part upon successful recruitment efforts to ensure adequate study power and a representative sample of participants that reflect the healthcare personnel population.

This paper addresses three objectives. First, we assess the representativeness of recruited participants relative to the source population of interest. Second, we evaluate the effect of each recruitment contact attempt in our protocol on study enrollment. Finally, we evaluate the prevalence of self-reported protective behaviors and potential exposures among healthcare personnel and determine which factors are associated with testing positive for SARS-CoV-2.

## Methods

### Multisite vaccine effectiveness study

The Centers for Disease Control and Prevention (CDC) led a 33-site study to evaluate the early effectiveness of a complete series of SARS-CoV-2 vaccines in preventing laboratory-confirmed, symptomatic COVID-19.^3,4^ The study used a test-negative case-control design. Cases were healthcare personnel with at least one symptom and a positive SARS-CoV-2 test. Week-matched controls were selected among personnel with a negative test result for SARS-CoV-2. Additional details of the study methods have previously been reported.^3,4^

### Utah study

The UU IRB approved the study at the UU Health. Healthcare personnel included all employees in clinical and patient-facing positions as well as support staff and other personnel within the healthcare setting. Employees were eligible if they sought polymerase chain reaction testing for SARS-CoV-2 at the UU Health from December 29, 2020 through July 31, 2021 and were symptomatic at time of testing. Exclusion criteria included a prior positive SARS-CoV-2 test, asymptomatic at time of testing, or participating in another UU COVID-19-related study.

Each week of the study, all SARS-CoV-2-positive symptomatic employees (cases) and three matched randomly selected symptomatic employees testing negative for SARS-CoV-2 (controls) were invited to participate. Vaccination status was ascertained using occupational health records, vaccine cards, or medical records. Vaccination status was classified in accordance with the multisite study as follows: fully vaccinated was defined as 7 or more days post second dose, partially vaccinated was defined as one dose or 6 or fewer days post second dose, and not vaccinated was defined as no vaccine doses.

### Recruitment and study procedures

In accordance with best practices,^5^ we developed a standardized recruitment protocol that included multiple reminders for nonrespondents and multiple modes of contact. If at any point during recruitment an employee completed the survey, they did not receive any additional messages. The following standard protocol was used for all potential participants with the exception noted below. Contact 1 was an email with a link to the online survey. Contact 2, three days later, was a text message reminder referring recipients to the first email message for information and the survey link. Contact 3, on Day 10, was a reminder email sent to nonrespondents. Nonrespondents were contacted by phone on Day 14 (Contact 4). We left a voicemail about the project for those who were not reached directly. If during the phone call the employee reported no longer having the email message on hand, we sent another email with a link to the survey that same day (Contact 5, second email reminder). All others who did not receive a reminder email on the same day as the phone call received Contact 5 at a later date if they still had not responded. A final reminder email was then sent to remaining nonrespondents (Contact 6). If at any point in this process, if an employee completed the consent form and began completing the survey, they were not subject to remaining reminder messages directed at nonrespondents. Alternatively, if they started the survey but did not complete it within two days of initiation, they received an automated reminder message asking them to complete their survey, but no further messages.

The recruitment email messages contained a link to a REDCap web survey. After confirming eligibility, individuals were asked to provide informed consent and then complete a questionnaire developed by the CDC. The questionnaire asked about COVID-19 testing, medical care related to current illness, comorbidities, demographics, vaccination, infection control practices, and potential COVID-19 exposures at work or outside of work. Potential exposures at work included close contact with an individual (either patient or non-patient) with confirmed or suspected COVID-19, regardless of personal protective equipment use. The survey also inquired about non-work potential exposures including close contact with sick individuals and participation in social activities.

### Data analysis

We calculated descriptive statistics for demographic characteristics of study participants. Individuals who participated in the study more than once were only included in analyses once, using data from their first enrollment. We compared demographics of participants to demographics of the study’s source population using chi-square tests. The source population data, provided by the University Electronic Data Warehouse, consisted of aggregate summary statistics representing the entire pool of employees who were tested during the study period, regardless of symptom presentation. These aggregate data were used because the study was not approved to retain demographic information on sampled nonparticipants.

Demographic variables included sex, ethnicity, race, age, and staff role. Staff role was categorized as providers (physicians, physician assistants, nurse practitioners), nursing (including assistants), allied healthcare workers (e.g., pharmacists, dieticians, social workers, physical therapists), administrative staff, support staff (e.g., customer service, facilities managers), other faculty, and other (including research and IT).

To evaluate the effect of the study protocol on response outcomes, we calculated the cumulative survey response proportion at each stage of the contact protocol, prior to each subsequent contact attempt, for cases, controls, and combined.

We assessed differences between cases and controls in protective behaviors and potential exposures using chi-square tests. We used conditional logistic regression to assess predictors of testing positive for SARS-CoV-2. A univariable model included vaccination status, and a multivariable model was conducted including variables exhibiting significant differences between cases and controls: vaccination, any potential exposure at work (composite variable representing contact via patient, coworker, or others), close contact to ill individuals outside work, and always wearing a mask outside of work (compared to less frequent wearing of masks). This multivariable model also adjusted for age, sex, race, Hispanic ethnicity, staff role (administrative vs. other), and presence of any underlying health condition known to increase severity of COVID-19.^3^ Conditional logistic regression was utilized to account for the weekly frequency-matched study design (3:1 controls to cases selected weekly).

## Results

### Demographic representativeness of study participants

Excluding 52 duplicate responses, 1456 employees were contacted, 608 initially consented, and 598 started the survey. A total of 503 (34.5%) employees enrolled and completed the survey (see Supplemental Figure: study flow). Participants were 76.7% female, 11.9% Hispanic or Latino, and 90.5% White (Table 1). Participants were younger with only 3.0% 60 years of age or older. Nurses (including registered nurses and nursing/medical assistants) comprised 31.6% of participants. We observed statistically significant differences between study participants and the source population in terms of sex, ethnicity, race, age, and staff role (Table 1). Because the university data warehouse race variable includes more race categories than were used in the study, race was indicated as “other” for 10.2% of the source population, whereas none of the study participants had race listed as “other” (*P* < 0.001). Participants were younger than the source population, and nursing staff comprised 31.6% of participants compared to 23.3% of the source population. There were no statistically significant differences in the demographics of enrolled cases compared to enrolled controls (see Supplemental Table).

**Table 1.** Demographics of study participants compared to source population for a study of COVID-19 vaccine effectiveness among healthcare personnel

	Study Participants n = 503		Source population^a^ n = 5,384		
	n	%	n	%	*P*
Sex					<0.001
Female	386	76.7	3834	71.2	
Male	114	22.7	1550	28.8	
Unknown/missing	3	0.6	0	0.0	
Ethnicity					<0.001
Hispanic or Latino	60	11.9	612	11.4	
Not Hispanic or Latino	439	87.3	4485	83.3	
Unknown/missing	4	0.8	287	5.3	
Race					<0.001
American Indian and Alaska Native	8	1.6	29	0.5	
Asian	24	4.8	291	5.4	
Black or African American	3	0.6	79	1.5	
Native Hawaiian and Pacific Islander	7	1.4	35	0.7	
White	455	90.5	4011	74.5	
Other	0	0	551	10.2	
Unknown/missing	20	4.0	388	7.2	
Age					<0.001
Under 30	151	30.0	871	16.2	
30–39	175	34.8	1783	33.1	
40–49	96	19.1	1273	23.6	
50–59	63	12.5	855	15.9	
60+	15	3.0	602	11.2	
Unknown/missing	3	0.6	0	0.0	
Staff role					<0.001
Administrative	76	15.1	866	16.1	
Allied healthcare workers	88	17.5	960	17.8	
Providers	65	12.9	739	13.7	
Nursing	159	31.6	1257	23.3	
Other	64	12.7	753	14.0	
Other faculty	10	2.0	333	6.2	
Support staff	36	7.2	476	8.8	
Unknown/missing	5	1.0	0	0.0	

a Source population data represents characteristics of eligible employees who were tested for Covid-19 during the study period.

### Effect of contact attempts on recruitment outcomes

We found that 23.9% of potential participants who received the standard contact protocol completed the survey after the initial invitation email, with no need for further contact attempts (Table 2). These early responders comprised over half of all total participants. A text message sent on day 3 produced 25 additional responses for a cumulative response rate of 26.0%. Each subsequent contact resulted in additional responses, with the final contact only resulting in three additional participants. Initially, participation was slightly higher among cases than controls, but the final response proportions were similar (33.6% of cases and 33.3% of controls). Of note, only 29% of our phone call attempts resulted in a live telephone conversation with the employee.

**Table 2.** Cumulative survey response by contact attempt, for cases, controls, and combined^a^

	Cases (test positive) n = 307		Controls (test negative) n = 861		Combined n = 1168	
Contact sequence^b^	Responses	Cumulative response %	Responses	Cumulative response %	Responses	Cumulative response %
1. Initial invite email (Day 1)	81	26.4	198	23.0	279	23.9
2. Text message (Day 3)	7	28.7	18	25.1	25	26.0
3. Follow-up email (Day 10)	3	29.6	16	27.0	19	27.7
4a. Phone call only (Day 14)	3	30.6	7	27.8	10	28.5
5a. 2^nd^ follow-up email (date varied, >14)	5	32.2	27	31.0	32	31.3
*4b. Phone call (Day 14) and 5b. 2^nd^ follow-up email (also Day 14)^c^*	*4*	*33.6*	*18*	*33.0*	*22*	*33.1*
6. 3^rd^ follow-up email (date varied)	0	33.6	3	33.3	3	33.4
Final response^d^	103	33.6	287	33.3	390	33.4

a Table includes only individuals who were subject to the contact protocol displayed in the table (the standard protocol). This table excludes individuals who began answering the survey but did not return to complete it for > 2 days, as they were not subject to the standard protocol after they had initiated the survey. Instead, if someone began the survey but did not complete it, after two days they were sent a reminder email to finish the survey. If they did not return to complete the survey, they received no further messages. Therefore, these individuals are excluded from counts in this table as this contact protocol was no longer applied to them.

b Once someone completed the survey, they did not receive any more recruitment messages.

c All nonrespondents received a phone call on Day 14. In this table we distinguish between those who received the contact 5 email after Day 14 (5a in table) and the subset of nonrespondents who received the phone call and the contact 5 email (contacts 4b and 5b) on the same day. These scenarios are documented separately in the table to reflect the fact that some individuals received two contacts in a single day, which could have influenced their likelihood of response differently than having received them on separate days.

d Final response count does not equate to 503 because table excludes individuals who did not receive the standard protocol.

### Association of potential exposures and protective behaviors with testing positive for SARS-CoV-2

Just over 28% of participants were fully vaccinated. Full vaccination was less common among cases (20.9%) than controls (30.8%, *P* = 0.01; Table 3). Among controls, 13.6% reported a potential COVID-19 exposure from a coworker, compared to only 4.7% of cases (*P* = 0.01). There were no significant differences between cases and controls in having close contact with a COVID-19 patient, mask-wearing frequency at work, or days per week employees worked in person on site. The percentage of cases who had close contact with someone ill outside of work (45.7%) was significantly higher than that among controls (20.1%; *P* < 0.001). Cases were slightly less likely to report always wearing a mask when outside of work (83.7% compared to 90.4%; *P* = 0.01). There were few significant differences between cases and controls in out-of-work behaviors with potential for exposure.

**Table 3.** Potential COVID-19 exposures at work and outside of work, for all participants and by case-control status

	All participants n = 503		Cases (test positive) n = 129		Controls (test negative) n = 374		
	n	%	n	%	n	%	*P*
Fully vaccinated	142	28.2	27	20.9	115	30.8	0.01
Partially vaccinated	163	32.4	36	27.9	127	34.0	
Not vaccinated	198	39.4	66	51.2	132	35.3	
Close contact with COVID-19 patient	107	21.3	23	17.8	84	22.5	0.27
Close contact at work, non-patient							0.01
Coworker	57	11.3	6	4.7	51	13.6	
Visitor	12	2.4	2	1.6	10	2.7	
Someone else	5	1.0	2	1.6	3	0.8	
Mask-wearing frequency at work							0.64
All of the time	425	84.5	108	83.7	317	84.8	
Most of the time	62	12.3	18	14.0	44	11.8	
Sometimes	5	1.0	0	0.0	5	1.3	
Rarely/never	11	2.2	3	2.3	8	2.1	
Days per week work in person							0.45
1	47	9.3	16	12.4	31	8.3	
2–3	131	26.0	34	26.4	97	25.9	
4–5	258	51.3	60	46.5	198	52.9	
6–7	17	3.4	5	3.9	12	3.2	
Close contact outside of work	134	26.6	59	45.7	75	20.1	<0.001
Behaviors with risk of exposure outside of work							
Used public transportation	63	12.5	17	13.2	46	12.3	0.80
Used rideshare	47	9.3	11	8.5	36	9.6	0.71
Household member attended school/daycare	174	34.6	41	31.8	133	35.6	0.44
Ate indoors at restaurant	217	43.1	65	50.4	152	40.6	0.05
Exercised at gym	91	18.1	25	19.4	66	17.6	0.66
Shop in store	434	86.3	110	85.3	324	86.6	0.70
Visited salon or barber	102	20.3	14	10.9	88	23.5	0.002
Attended gathering < 10 people	260	51.7	57	44.2	203	54.3	0.05
Attended gathering > 10 people	78	15.5	19	14.7	60	16.0	0.72
Mask-wearing frequency outside of work							0.01
Always	446	88.7	108	83.7	338	90.4	
Sometimes	40	8.0	15	11.6	25	6.7	
Rarely	14	2.8	3	2.3	11	2.9	
Never	3	0.6	3	2.3	0	0.0	

Full vaccination was associated with significantly lower odds of testing positive for SARS-CoV-2 (OR: 0.20; 95% CI: 0.08, 0.46; Table 4). This equates to vaccine effectiveness of 80% (1.0 – 0.20 = 0.80). The odds of testing positive for SARS-CoV-2 among partially vaccinated individuals was 0.62 (95% CI: 0.37, 1.03; vaccine effectiveness: 38%). In the multivariable model, those with contact with someone ill outside of work had 3.74 higher odds of testing positive for SARS-CoV-2 than those who did not (OR = 3.74; 95% CI: 2.29, 6.11). Conversely, potential exposure at work was inversely associated with testing positive for SARS-CoV-2 (OR: 0.51, 95% CI: 0.29, 0.88) as was being fully vaccinated for COVID-19 (OR: 0.21; 95% CI: 0.08, 0.52). Frequency of mask-wearing outside of work was not significantly associated with a positive test, nor were any participant characteristics or underlying health conditions.

**Table 4.** Conditional logistic regression predicting positive test for SARS-CoV-2 among healthcare personnel^a^

	Model 1				Model 2^b^			
	OR	95% CI		*P*	OR	95% CI		*P*
Vaccination status								
Fully vaccinated	0.20	0.08, 0.46		<0.001	0.21	0.08, 0.52		<0.001
Partially vaccinated	0.62	0.37, 1.03		0.07	0.62	0.36, 1.09		0.10
Not vaccinated	Ref				Ref			
Potential COVID-19 exposure at work								
Yes	–				0.51	0.29, 0.88		0.02
No	Ref				Ref			
Potential exposure outside of work								
Yes	–				3.74	2.29, 6.11		<0.001
No	Ref				Ref			
Mask-wearing outside work								
Always	–				0.53	0.23, 1.25		0.15
Sometimes, rarely, never	Ref				Ref			

Note. OR, Odds ratio; CI, Confidence interval; Ref, referent category.

a Conditional logistic regression was used to account for study design using weekly frequency-matched cases and controls.

b Model 2 adjusts for employee age, sex, race, Hispanic ethnicity, staff role, and presence of any underlying health condition.

## Discussion

Healthcare facilities still grapple with COVID-19 infections among employees. This study evaluated factors associated with SARS-CoV-2 infection among healthcare personnel. Results can inform efforts to prevent future outbreaks among healthcare personnel. By assessing the effectiveness of our recruitment protocol and the representativeness of participants, this study adds insight into successes and challenges healthcare organizations face recruiting healthcare personnel in challenging circumstances such as a global pandemic.

When initiating this study, we recognized that healthcare personnel had been substantially impacted by the pandemic, including burnout and mental health effects,^6,7^ experiencing COVID-19-related bullying and harassment,^8^ and changes to work hours, duties, and job security.^9^ The proliferation of surveys during the COVID-19 pandemic has increased survey fatigue and reduced participation among healthcare personnel.^10^ Thus, we sought to assess how well our recruitment protocol fared.

Our final response proportion, 34.5%, exceeds that of some COVID-19-related studies^11^ but is similar to others.^12,13^ Over half of the individuals who participated did so after one contact attempt, with no reminder messages needed. However, each reminder message in our protocol added additional participants. Without subsequent contacts, our final response would have been approximately 10 percentage points lower, and we would have had fewer controls in the study. We conclude that each contact was worthwhile except for the sixth contact, which only yielded three additional responses. It is often difficult to recruit controls for case-control studies.^14^ This study demonstrates that multiple reminders increase recruitment of controls.

The added value of our reminder messages is consistent with prior survey research,^15^ including among healthcare professionals.^16,17^ Multiple modes of contact may add novelty to a different stimulus.^18^ Email messages were the most effective in increasing the number of participants, likely because the emails were the only contacts with a direct link to the online survey. Study team members making phone calls reported that their conversations with prospective participants appeared helpful in encouraging participation. However, less than one-third of call attempts resulted in reaching the person live, which is consistent with contact rates reported for national public opinion research.^19^

The demographics of enrolled participants differed from the source population The use of a different measure of race (with fewer categories) in the study compared to what is used by the university resulted in substantially more reports of “other” races in the source population than in the study. The overrepresentation of younger individuals in our study was surprising, as young age is often associated with nonparticipation and underrepresentation in research.^20,21^ Nursing staff were overrepresented in the study sample relative to other staff roles. We found no significant differences in the demographics of cases compared to controls enrolled in the study, assuring that any potential bias in demographic representativeness was not unevenly affecting cases compared to controls.

The negative association between potential COVID-19 exposure at work and testing positive for SARS-CoV-2 was unexpected. One possibility is that potential workplace exposures were not as risky as outside exposures due to the ample supply, and protocols for use, of personal protective equipment in the workplace. It is also possible that the negative relationship between potential work exposure to COVID-19 and testing positive is indicative of testing bias in the study. Test-negative designs can be susceptible to bias if the exposure of interest differentially influences the propensity to test in cases versus controls.^22^ Thus, the bias could arise if test-seeking was influenced both by work-related contact and by the likelihood that COVID-19 is the cause of symptoms. Such a situation would occur if a work-related exposure generally led to testing, regardless of clinical presentation, whereas having a clinical picture more specific for COVID-19 increased testing among individuals who did not have a work-related exposure. Consistent with this hypothesis, the apparent protective effect of work-related potential exposure was diminished (and no longer statistically significant) in a model that included fatigue and alteration in smell or taste, two symptoms that were associated with test positivity. The policy at UU Health was that employees with close contact with a coworker testing positive for SARS-CoV-2 were contacted by Employee Health and encouraged to seek testing. A bias in the opposite direction could arise if vaccinated individuals were less likely to seek testing than non-vaccinated individuals. However, we did not find any evidence that adjusting for presence of symptoms modified our estimate of vaccine effectiveness.

Research has found a variety of factors are associated with higher SARS-CoV-2 seropositivity among healthcare workers, including patient-facing positions, front-line COVID-19 healthcare positions, and shortages or lack of use of personal protective equipment.^23^ We found that a potential exposure to someone ill outside of work, not potential exposure at work, was the primary predictor of testing positive for SARS-CoV-2. Others have reported the significance of community exposures, not workplace exposures, for predicting infection in healthcare personnel.^24,25^ These findings could also suggest that infection control measures in healthcare settings are effective at preventing transmission.^26^

This study is subject to limitations. Our analysis is reliant on participants’ self-reports of their protective behaviors and possible exposures. We were unable to retain demographic data on sampled personnel who declined to participate in the study for purposes of assessing the demographic representativeness of the study participants. Thus, we were limited to using aggregate data obtained from the Electronic Data Warehouse after the study conclusion. This prevented us from assessing representativeness of the sample by week of recruitment. Our study was conducted only in English, which may have been a barrier to participation among some personnel.

This study provides multiple contributions. Our assessment of the effectiveness of recruitment attempts on enrolling healthcare personnel in a research study during a pandemic should guide future researchers in efforts to recruit healthcare professionals. Our results show the value of a multiple contact, multimode approach for increasing study participation. Second, we demonstrated that demographic characteristics are associated with study participation, but that there was no significant difference in demographic characteristics between cases and controls. Our analyses add to the literature showing that among healthcare personnel, potential exposures outside of the healthcare setting are more strongly related to testing positive for SARS-CoV-2 than potential exposures within the healthcare setting. This finding demonstrates the difficulty healthcare facilities face in preventing outbreaks among employees, as it is not possible to control community exposures. Hospitals must emphasize to personnel the ongoing risk of SARS-CoV-2 infection in the community and encourage continued testing.

## Supporting information

Millar et al. supplementary material 1 Millar et al. supplementary material

Millar et al. supplementary material 2 Millar et al. supplementary material

## Supplementary material

The supplementary material for this article can be found at https://doi.org/10.1017/ash.2024.44

## References

[1] U.S. Food and Drug Administration. FDA takes key action in fight against COVID-19 by issuing Emergency Use Authorization for first COVID-19 vaccine. https://www.fda.gov/news-events/press-announcements/fda-takes-key-action-fight-against-covid-19-issuing-emergency-use-authorization-first-covid-19. Published 2020. Accessed September 29, 2022

[2] Dooling K , McClung N , Chamberland M , Marin M , Wallace M , Bell BP , Lee GM , Talbot HK , Romero JR , Oliver SE. The advisory committee on immunization practices’ interim recommendation for allocating initial supplies of COVID-19 vaccine - United States, 2020. MMWR Morb Mortal Wkly Rep 2020;69:1857–1859. 33301429 10.15585/mmwr.mm6949e1 PMC7737687

[3] Pilishvili T , Gierke R , Fleming-Dutra KE , Farrar JL , Mohr NM , Talan DA , Krishnadasan A , Harland KK , Smithline HA , Hou PC , Lee LC. Effectiveness of mRNA Covid-19 vaccine among U.S. health care personnel. N Engl J Med 2021;385:e90. 34551224 10.1056/NEJMoa2106599 PMC8482809

[4] Pilishvili T , Fleming-Dutra KE , Farrar JL , et al. Interim estimates of vaccine effectiveness of Pfizer-BioNTech and Moderna COVID-19 vaccines among health care personnel - 33 U.S. Sites, January-March 2021. *MMWR Morb Mortal Wkly Rep* 2021;70:753–758. 10.15585/mmwr.mm7020e2 PMC8136422 34014909

[5] Dillman DA , Smyth JD , Christian LM. Internet, Phone, Mail, and Mixed-Mode Surveys: The Tailored Design Method, 4th edition. Hoboken, NJ: Wiley; 2014.

[6] Danet Danet A. Psychological impact of COVID-19 pandemic in Western frontline healthcare professionals. A systematic review. *Med Clin (Barc)* 2021;156:449–458. Impacto psicológico de la COVID-19 en profesionales sanitarios de primera línea en el ámbito occidental. Una revisión sistemática. 10.1016/j.medcli.2020.11.009 PMC7775650 33478809

[7] Batra K , Singh TP , Sharma M , Batra R , Schvaneveldt N. Investigating the psychological impact of COVID-19 among healthcare workers: a meta-analysis. *Int J Environ Res Public Health* 2020;17:9096. 10.3390/ijerph17239096 PMC7730003 33291511

[8] Dye TD , Alcantara L , Siddiqi S , Barbosu M , Sharma S , Panko T , Pressman E. Risk of COVID-19-related bullying, harassment and stigma among healthcare workers: an analytical cross-sectional global study. BMJ Open 2020;10:e046620. 10.1136/bmjopen-2020-046620 PMC7780430 33380488

[9] Keihanian T , Sharma P , Sandhu DS , Sussman DA , Tabibian JH , Girotra M. Impact of the COVID-19 pandemic on clinical schedules and physical and mental well-being of gastroenterology nonphysician healthcare workers: a nationwide survey. Gastroenterol Nurs 2021;44:240–251. 34149038 10.1097/SGA.0000000000000599 PMC8318563

[10] de Koning R , Egiz A , Kotecha J , Ciuculete AC , Ooi SZ , Bankole ND , Erhabor J , Higginbotham G , Khan M , Dalle DU , Sichimba D. Survey fatigue during the COVID-19 pandemic: an analysis of neurosurgery survey response rates. Front Surg 2021;8:690680. 34458314 10.3389/fsurg.2021.690680 PMC8388838

[11] Oberleitner LMS , Lucia VC , Navin MC , Ozdych M , M. Afonso N , Kennedy RH , Keil H , Wu L , Mathew TA. COVID-19 vaccination concerns and reasons for acceptance among US health care personnel. Public Health Rep 2022;137:1227–1234. 36073241 10.1177/00333549221120590 PMC9459372

[12] Byhoff E , Paulus JK , Guardado R , Zubiago J , Wurcel AG. Healthcare workers’ perspectives on coronavirus testing availability: a cross sectional survey. BMC Health Serv Res 2021;21:719. 34289840 10.1186/s12913-021-06741-5 PMC8294832

[13] Momplaisir FM , Kuter BJ , Ghadimi F , Browne S , Nkwihoreze H , Feemster KA , Frank I , Faig W , Shen AK , Offit PA , Green-McKenzie J. Racial/ethnic differences in COVID-19 vaccine hesitancy among health care workers in 2 large academic hospitals. JAMA Netw Open 2021;4:e2121931. 34459907 10.1001/jamanetworkopen.2021.21931 PMC8406078

[14] Moorman PG , Newman B , Millikan RC , Tse CK , Sandler DP. Participation rates in a case-control study: the impact of age, race, and race of interviewer. Ann Epidemiol 1999;9:188–195. 10192651 10.1016/s1047-2797(98)00057-x

[15] Edwards PJ , Roberts I , Clarke MJ , Diguiseppi C , Wentz R , Kwan I , Cooper R , Felix LM , Pratap S. Methods to increase response to postal and electronic questionnaires. *Cochrane Database Syst Rev* 2009;3:MR000008. 10.1002/14651858.MR000008.pub4 PMC8941848 19588449

[16] Cho YI , Johnson TP , VanGeest JB. Enhancing surveys of health care professionals: a meta-analysis of techniques to improve response. Eval Health Prof 2013;36:382–407. 23975761 10.1177/0163278713496425

[17] Meyer VM , Benjamens S , Moumni ME , Lange JFM , Pol RA. Global overview of response rates in patient and health care professional surveys in surgery: a systematic review. Ann Surg 2022;275:e75–e81. 32649458 10.1097/SLA.0000000000004078 PMC8683255

[18] de Leeuw ED. To mix or not to mix data collection modes in surveys. J Stat 2005;21:233–255.

[19] Marken S. Still listening: The state of telephone surveys. https://news.gallup.com/opinion/methodology/225143/listening-state-telephone-surveys.aspx. Published 2018. Accessed December 28, 2023.

[20] Beebe TJ , McAlpine DD , Ziegenfuss JY , Jenkins S , Haas L , Davern ME. Deployment of a mixed-mode data collection strategy does not reduce nonresponse bias in a general population health survey. Health Serv Res 2012;47:1739–1754. 22250782 10.1111/j.1475-6773.2011.01369.x PMC3330173

[21] Schneider KL , Clark MA , Rakowski W , Lapane KL. Evaluating the impact of non-response bias in the Behavioral Risk Factor Surveillance System (BRFSS). J Epidemiol Community Health 2012;66:290–295. 20961872 10.1136/jech.2009.103861

[22] Lewnard JA , Tedijanto C , Cowling BJ , Lipsitch M. Measurement of vaccine direct effects under the test-negative design. Am J Epidemiol 2018;187:2686–2697. 30099505 10.1093/aje/kwy163 PMC6269249

[23] Galanis P , Vraka I , Fragkou D , Bilali A , Kaitelidou D. Seroprevalence of SARS-CoV-2 antibodies and associated factors in healthcare workers: a systematic review and meta-analysis. J Hosp Infect 2021;108:120–134. 33212126 10.1016/j.jhin.2020.11.008 PMC7668234

[24] Jacob JT , Baker JM , Fridkin SK , Lopman BA , Steinberg JP , Christenson RH , King B , Leekha S , O’Hara LM , Rock P. Risk factors associated with SARS-CoV-2 seropositivity among US health care personnel. JAMA Netw Open 2021;4:e211283. 33688967 10.1001/jamanetworkopen.2021.1283 PMC7948059

[25] Kobayashi T , Trannel A , Heinemann J , Marra AR , Etienne W , Abosi OJ , Holley S , Dains A , Jenn KE , Meacham H , Schuessler BA , Wendt L , Ten Eyck P , Hanna B , Salinas JL , Hartley PG , Ford B , Wellington M , Brust KB , Diekema DJ . Association between job role and coronavirus disease 2019 (COVID-19) among healthcare personnel, Iowa, 2021. *Antimicrob Steward Healthc Epidemiol* 2022;2:e188. 10.1017/ash.2022.349 PMC9726628 36505945

[26] Hori H , Fukuchi T , Sanui M , Moriya T , Sugawara H. Comprehensive infection control measures prevent hospital-acquired severe acute respiratory syndrome coronavirus 2 infection: A single-center prospective cohort study and seroprevalence survey. PLoS One 2021;16:e0257513. 34634076 10.1371/journal.pone.0257513 PMC8504754

