# Supplementary material for: Factors associated with testing positive for SARS-CoV-2 and evaluation of a recruitment protocol among healthcare personnel in a COVID-19 vaccine effectiveness study
Source: Antimicrob Steward Healthc Epidemiol. 2024 Apr 16;4(1):e47. doi: 10.1017/ash.2024.44 (PMC11019576; doi:10.1017/ash.2024.44)
Supplement: Millar et al. supplementary material 1 — Millar et al. supplementary material [file S2732494X24000445sup001.docx]

**Supplemental Table. Demographics of cases and controls**

|  | Cases (test-positive) | | | | | Controls (test-negative) | | | | | | |  |
| --- | --- | --- | --- | --- | --- | --- | --- | --- | --- | --- | --- | --- | --- |
|  | Study participants n=129 | | | Source population n=462 | | Study participants n=374 | | | Source population n=4,922 | | | |  |
|  | n | % | n | | % | | n | % | | n | % | *p*^a^ | |
| Sex |  |  |  | |  | |  |  | |  |  | 0.07 | |
| Female | 92 | 71.3% | 328 | | 71.0% | | 294 | 78.6% | | 3506 | 71.2% |  | |
| Male | 37 | 28.7% | 134 | | 29.0% | | 77 | 20.6% | | 1416 | 28.8% |  | |
| Unknown/missing | 0 | 0.0% | 0 | | 0.0% | | 3 | 0.8% | | 0 | 0.0% |  | |
|  |  |  |  | |  | |  |  | |  |  |  | |
| Ethnicity |  |  |  | |  | |  |  | |  |  | 0.41 | |
| Hispanic/Latino | 18 | 14.0% | 73 | | 15.8% | | 42 | 11.2% | | 539 | 11.0% |  | |
| Not Hispanic/Latino | 110 | 85.3% | 365 | | 79.0% | | 329 | 88.0% | | 4120 | 83.7% |  | |
| Unknown/missing | 1 | 0.8% | 24 | | 5.2% | | 3 | 0.8% | | 263 | 5.3% |  | |
|  |  |  |  | |  | |  |  | |  |  |  | |
| Race |  |  |  | |  | |  |  | |  |  | 0.246 | |
| American Indian/Alaska Native | 3 | 2.3% | 6 | | 1.3% | | 5 | 1.3% | | 23 | 0.5% |  | |
| Asian | 3 | 2.3% | 12 | | 2.6% | | 21 | 5.6% | | 279 | 5.7% |  | |
| Black or African American | 2 | 1.6% | 11 | | 2.4% | | 1 | 0.3% | | 68 | 1.4% |  | |
| Native Hawaiian/Pacific Islander | 2 | 1.6% | 4 | | 0.9% | | 5 | 1.3% | | 31 | 0.6% |  | |
| White | 121 | 93.8% | 330 | | 71.4% | | 334 | 89.3% | | 3681 | 74.8% |  | |
| Other | 0 | 0.0% | 62 | | 13.4% | | 0 | 0.0% | | 489 | 9.9% |  | |
| Unknown/missing | 4 | 3.1% | 37 | | 8.0% | | 16 | 4.3% | | 351 | 7.1% |  | |
|  |  |  |  | |  | |  |  | |  |  |  | |
| Age |  |  |  | |  | |  |  | |  |  | 0.98 | |
| Under 30 | 39 | 30.2% | 90 | | 19.5% | | 112 | 29.9% | | 781 | 15.9% |  | |
| 30-39 | 44 | 34.1% | 152 | | 32.9% | | 131 | 35.0% | | 1631 | 33.1% |  | |
| 40-49 | 26 | 20.2% | 119 | | 25.8% | | 70 | 18.7% | | 1154 | 23.4% |  | |
| 50-59 | 16 | 12.4% | 71 | | 15.4% | | 47 | 12.6% | | 784 | 15.9% |  | |
| 60+ | 3 | 2.3% | 30 | | 6.5% | | 12 | 3.2% | | 572 | 11.6% |  | |
| Unknown/missing | 1 | 0.8% | 0 | | 0.0% | | 2 | 0.5% | | 0 | 0.0% |  | |
|  |  |  |  | |  | |  |  | |  |  |  | |
| Staff role |  |  |  | |  | |  |  | |  |  | 0.85 | |
| Administrative | 22 | 17.1% | 73 | | 15.8% | | 54 | 14.4% | | 793 | 16.1% |  | |
| Allied Healthcare Workers | 19 | 14.7% | 88 | | 19.0% | | 69 | 18.4% | | 872 | 17.7% |  | |
| Providers | 15 | 11.6% | 46 | | 10.0% | | 50 | 13.4% | | 693 | 14.1% |  | |
| Nursing | 42 | 32.6% | 117 | | 25.3% | | 117 | 31.3% | | 1140 | 23.2% |  | |
| Other | 20 | 15.5% | 72 | | 15.6% | | 44 | 11.8% | | 681 | 13.8% |  | |
| Other Faculty | 2 | 1.6% | 13 | | 2.8% | | 8 | 2.1% | | 320 | 6.5% |  | |
| Support Staff | 9 | 7.0% | 53 | | 11.5% | | 27 | 7.2% | | 423 | 8.6% |  | |
| Unknown/missing | 0 | 0.0% | 0 | | 0.0% | | 5 | 1.3% | | 0 | 0.0% |  | |

1. P-values from chi-square tests comparing enrolled cases to enrolled controls.
