# Supplementary figures and images for: Factors associated with testing positive for SARS-CoV-2 and evaluation of a recruitment protocol among healthcare personnel in a COVID-19 vaccine effectiveness study
Source: Antimicrob Steward Healthc Epidemiol. 2024 Apr 16;4(1):e47. doi: 10.1017/ash.2024.44 (PMC11019576; doi:10.1017/ash.2024.44)

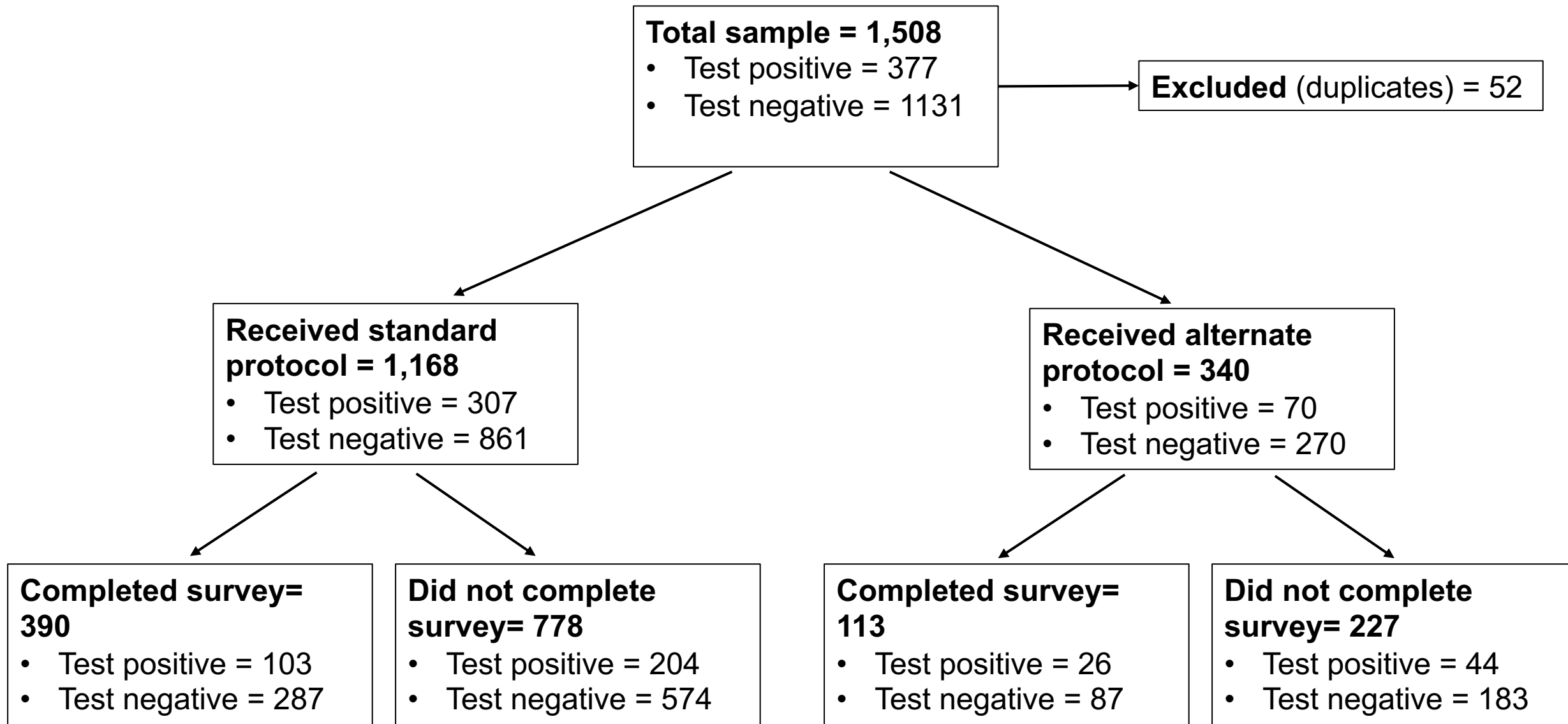

**Supplemental Figure 1. Study Flow**

Supplement: Millar et al. supplementary material 2 — Millar et al. supplementary material [file S2732494X24000445sup002.pdf]
